# Tai Chi as a Mind–Body Intervention to Address Chronic Pain in Socially Isolated Older Adults: A Narrative Review

**DOI:** 10.3390/healthcare14111464

**Published:** 2026-05-26

**Authors:** Nina H. Russin, Matthew P. Martin

**Affiliations:** College of Health Solutions, Arizona State University, Health North, 550 N. 3rd St., Mail Code 9020, Phoenix, AZ 85004, USA

**Keywords:** chronic pain, social isolation, loneliness, older adults, elderly, geriatric, Tai Chi

## Abstract

**Background:** Chronic pain affects approximately 30% of older adults and is strongly associated with social isolation and loneliness, which impact an estimated 25% of the global older adult population. A substantial proportion of chronic pain in this population is classified as primary chronic pain (non-specific), characterized by persistent pain with no underlying disease or structural damage. Pharmacotherapy has limited efficacy in treating primary chronic pain and presents significant polypharmacy risks, highlighting a critical need for sustainable, non-pharmacologic interventions. Among these, Tai Chi has emerged as a promising multimodal therapy, it is a mind–body exercise that integrates gentle movement and focused breathing with social engagement, offering participants both physical relief and opportunities for meaningful human connection. Gentle movement for flexibility, balance, and strength, combined with deep breathing may also improve self-reported symptoms of chronic pain, in addition to inflammatory biomarkers such as *CRP*, *IL-6* and *TNFα*. The purpose of this narrative review is to investigate the literature on Tai Chi as a method for promoting socialization and reducing self-rated chronic pain among community-dwelling, socially isolated older adults. **Methods:** Following librarian-assisted concept map development, we searched six electronic databases (PubMed, CINAHL, Scopus, Cochrane, ProQuest, and PsycINFO) for studies published between January 2016 and February 2026. Search strings included terms for “older adults,” “chronic pain,” “social isolation/loneliness,” and “Tai Chi.” Two reviewers independently screened results and extracted data for relevance. **Results:** Of the 1098 records identified, 25 studies met the inclusion criteria. Eleven studies evaluated Tai Chi or related mind–body interventions. Among these, approximately six studies reported improvements in pain-related outcomes, while five studies reported improvements in loneliness or social isolation. However, only two to three studies simultaneously evaluated both chronic pain and social isolation/loneliness outcomes within Tai Chi interventions. Overall, most studies supported Tai Chi as a safe and potentially effective intervention for older adults, with evidence suggesting benefits for both pain and social well-being. However, the limited number of studies examining combined outcomes restricts conclusions regarding the integrated effects of Tai Chi on chronic pain and social isolation. **Discussion:** Tai Chi is a safe, inexpensive behavioral strategy for improving social connectedness and reducing self-rated chronic pain among older adults. However, the evidence base remains fragmented, as pain and social isolation are rarely assessed together within the same trial. Future research should address this gap by considering both social isolation and chronic pain in the same study, with more standardized Tai Chi forms as the single independent variable.

## 1. Introduction

Chronic pain affects approximately 30% of older adults and is strongly associated with social isolation and loneliness, which impact an estimated 25% of the global older adult population [1,2,3,4]. A substantial proportion of chronic pain in this population is classified as chronic primary pain, characterized by persistent pain with no underlying disease or structural damage. The International Association for the Study of Pain (IASP) defines chronic pain as pain lasting longer than the usual recovery time from an injury or illness, typically beyond 3–6 months, and which is either persistent or intermittent [5,6]. Pharmacotherapy has limited usefulness in treating primary chronic pain, particularly in older adults, where concerns include drug–drug interactions and polypharmacy, pointing to a need for sustainable, non-pharmacologic approaches.

Particularly in older adults, chronic pain has been associated with social isolation, defined as the lack of social contact or support, and loneliness, which is a subjective feeling of being alone or isolated [7]. In China, where chronic pain is estimated to affect up to 40% of older adults, a study based on three waves of the Chinese Health and Retirement Longitudinal Study (CHARLS) found that both baseline social isolation and loneliness were associated with a higher likelihood of chronic pain [8]. Similar correlations between loneliness, social isolation and chronic pain have been documented in studies in the United States [9], the United Kingdom [10], and Japan [11].

Older adults experiencing both chronic pain and loneliness or social isolation present a particular treatment challenge, as interventions must address physical symptoms, psychological well-being, and social connectedness simultaneously. From a population health perspective, identifying non-pharmacologic interventions that are accessible, acceptable, and feasible for older adults with potentially limited healthcare access is especially important.

Several psychosocial interventions, including cognitive behavioral therapy (CBT) and related “third-wave” approaches such as mindfulness-based stress reduction and acceptance and commitment therapy, may improve both chronic pain and loneliness outcomes [12,13]. However, these interventions are frequently delivered within clinical settings and may require ongoing access to trained behavioral health professionals. In addition, a recent systematic review and meta-analysis evaluating CBT interventions for loneliness among older adults reported substantial heterogeneity across studies and concluded that evidence of efficacy remains exploratory rather than definitive [14].

Other complementary approaches, including acupuncture, hypnosis, and biofeedback, may be beneficial for certain chronic pain conditions. However, these modalities are typically delivered through one-to-one patient–provider interactions and are not primarily designed to promote social engagement or social network development. These approaches may also require specialized training, licensure, or repeated clinical visits, potentially limiting accessibility for some older adults.

By comparison, Tai Chi is a community-based mind–body practice that combines gentle physical activity, mindfulness, and group participation. Although Tai Chi originated within Chinese martial arts traditions [15], its emphasis on controlled movement, balance, and adaptability has contributed to its growing use among older adults and individuals with chronic pain. Findings from the present review suggest that Tai Chi may offer benefits across both physical and social domains, with several studies reporting improvements in pain outcomes, perceived social support, loneliness, or quality of life. These characteristics may make Tai Chi particularly well-suited for older adults experiencing chronic pain alongside social isolation or loneliness.

Tai Chi derives its name from the concepts of yin and yang: two opposite forces that are interconnected and interdependent in the world. Physically, Tai Chi seeks to integrate the left and right halves of the body through stretching, strengthening, balance, and coordination. Tai Chi is also a mindful practice that integrates gentle, physical movements with rhythmic breathing, focused attention, and present-moment awareness [15]. Tai Chi classes offer opportunities for meaningful human connections [16]. Gentle movement for flexibility, balance and strength, combined with deep breathing may also improve self-reported symptoms of chronic pain, in addition to inflammatory biomarkers such as *CRP*, *IL-6* and *TNFα* [17].

Although there are several effective treatments for chronic pain and social isolation, there is limited guidance for clinicians selecting interventions that simultaneously treat both conditions among older adults. The purpose of this narrative review is to investigate the literature on Tai Chi as a method for promoting socialization and reducing self-rated chronic pain among community-dwelling, socially isolated older adults. Tai Chi’s widespread popularity is supported by estimates of 300 million individuals practicing various forms of Tai Chi globally, including 1.5 million in the United States alone [18]. Tai Chi has been described as a socially and culturally sustainable practice [18], with benefits including emotional regulation, social connectedness, and chronic pain reduction. This reflects the biopsychosocial model of chronic pain, which assumes a major psychological influence [19].

## 2. Methods

### 2.1. Study Design

This study was conducted as a narrative review to synthesize recent evidence on the relationship between Tai Chi, chronic pain, and social isolation or loneliness among older adults. Unlike systematic or scoping reviews, this approach was intended to provide a targeted, interpretive synthesis of selected literature rather than a comprehensive review of all available evidence. A narrative review design was selected because relatively few studies simultaneously examined all three constructs, requiring integration of evidence across heterogeneous study designs, populations, and outcome measures.

### 2.2. Search Strategy

Prior to initiating the search, the first author, a Doctor of Behavioral Health with expertise in chronic pain, collaborated with a university science librarian to develop a concept map and search strategy. The initial search was conducted by the first author and subsequently reviewed independently by the second author, a PhD-trained medical family therapist. The science librarian’s assistance was crucial in creating targeted and unbiased search strategies. In the event of a conflict of opinion, the first author deferred to the second author, given his more extensive clinical and research experience.

The following databases were searched: PubMed, APA, PsycINFO^®^, Scopus, Cochrane Database of Systematic Reviews, CINAHL, and ProQuest (via institutional library access). Search terms included combinations of the following keywords: (“older adults” OR “elderly” OR “geriatric”) AND “chronic pain” AND (“social isolation” OR “loneliness”) AND “Tai Chi.” Titles and abstracts were screened for relevance, followed by full-text review to determine inclusion.

### 2.3. Eligibility Criteria

Inclusion criteria were: (1) peer-reviewed articles published in English; (2) studies published between 1 January 2016, and 28 February 2026; and (3) studies examining at least one of the following domains: chronic pain, social isolation or loneliness, and/or Tai Chi or related mind–body interventions in older adult populations. Exclusion criteria included gray literature (e.g., dissertations, conference abstracts, and unpublished reports).

### 2.4. Study Selection and Review Process

Titles and abstracts were screened for relevance by the first author, followed by full-text reviews to determine eligibility. Study selection decisions were independently reviewed by the second author, and discrepancies regarding inclusion were resolved through discussion and consensus.

### 2.5. Narrative Synthesis Approach

Given the limited number of studies simultaneously examining Tai Chi, chronic pain, and social isolation or loneliness, studies addressing related psychosocial and contextual dimensions were also included. Priority was given to larger population-based studies and systematic reviews when available. However, qualitative studies, pilot studies, and smaller community-based investigations were retained when they provided insight into underrepresented populations, participant experiences, or factors influencing long-term engagement with Tai Chi interventions. This approach enabled synthesis of both quantitative outcomes and qualitative findings relevant to the biopsychosocial context of chronic pain among older adults.

### 2.6. Methodological Considerations

Significant heterogeneity existed across included studies, including differences in Tai Chi forms, intervention duration and frequency, instructor experience, participant characteristics, recruitment strategies, and outcome measures. Many studies and reviews did not specify the exact Tai Chi form or intervention structure utilized. Variability in study design, including inconsistent use of control groups and follow-up periods, limited direct comparability across studies. Accordingly, findings should be interpreted as primarily associative rather than causal. Formal risk-of-bias assessment was not conducted because this study was designed as a narrative rather than a systematic review.

## 3. Results

**Summary of Evidence**. Of the 1098 records identified, 25 studies met the inclusion criteria. The included studies represented a diverse global sample, with the majority conducted in the United States (n = 8), China (n = 5), and Japan (n = 4), along with studies from the United Kingdom (n = 1), Sweden (n = 1), Belgium (n = 1), and Iran (n = 1). Four studies did not specify a geographic origin, as they were reviews.

Study designs were heterogeneous and included cross-sectional studies (n = 7), longitudinal or cohort studies (n = 5), qualitative studies (n = 3), randomized or non-randomized controlled trials (n = 1), mixed-methods studies (n = 1), and reviews (systematic reviews and meta-analyses, n = 5; narrative reviews, n = 2). Sample sizes varied substantially, ranging from 18 to 21,463 participants, with several large population-based datasets included.

Across studies, the predominant population was older adults, typically aged 60 years and older, although some studies included participants aged 50+. Most studies included both male and female participants, with several demonstrating a higher proportion of female participants, reflecting demographic trends in aging populations.

Across all included studies, key variables examined included chronic pain (n = 18 studies), loneliness (n = 15), social isolation (n = 14), and Tai Chi or related mind–body interventions (n = 11). Table 1 summarizes the 25 included studies, including geographic origin, study design, sample size, and key demographic characteristics (age and sex distribution, where available), along with relevant population descriptors. Table 2 synthesizes the included studies by study focus, key variables, and main findings.

### 3.1. Loneliness, Social Isolation, and Chronic Pain

Several large cohort studies demonstrated that loneliness was associated with an increased likelihood and severity of chronic pain [8,10,11]. In longitudinal analyses, loneliness increased both prior to and following the onset of chronic pain [10]. Similarly, social isolation and lack of social participation were associated with increased disability risk among individuals with chronic pain [30].

Cross-sectional studies further supported these findings, demonstrating associations between loneliness and pain presence, intensity, and interference [9,20]. In some populations, perceived social support moderated the relationship between pain intensity and functional outcomes [20].

Several studies also reported associations between loneliness and broader health outcomes, including depression, sleep disturbances, and cognitive impairment [25,31]. Notably, some studies found that loneliness and social isolation exerted independent and, in some cases, synergistic effects on chronic pain outcomes [11].

### 3.2. Tai Chi and Chronic Pain Outcomes

Interventional and review studies generally supported the effectiveness of Tai Chi in reducing self-reported pain among older adults [14,21,27]. However, findings were not uniformly consistent. One qualitative study reported mixed results for chronic low back pain, although participants described improvements in physical function and well-being [22].

### 3.3. Tai Chi and Social Outcomes

These studies reported improvements in perceived social support, reduced loneliness, and enhanced social engagement among participants in community-based Tai Chi programs [13,21,28]. Qualitative findings further suggested that group-based Tai Chi participation fostered interpersonal connection and emotional well-being [32].

### 3.4. Combined Effects of Tai Chi on Pain and Social Outcomes

Only a small subset of studies (n = 2–3) simultaneously evaluated both chronic pain and social isolation or loneliness outcomes within Tai Chi interventions. These studies suggested potential dual benefits but were limited by small sample sizes or qualitative designs [13,32]. Future research should prioritize larger studies, including randomized controlled trials, that simultaneously assess pain, loneliness, and social participation outcomes to better understand these interrelated pathways. This could be particularly impactful when considering collectivistic cultures in Asia and Native American communities.

### 3.5. Physiological Mechanisms

Evidence regarding physiological mechanisms was limited. A small number of studies examined biomarkers, suggesting that Tai Chi may influence endogenous opioid pathways and inflammatory processes [31]. However, mechanistic evidence remains sparse.

## 4. Discussion

This review identified consistent evidence linking loneliness and social isolation with chronic pain outcomes among older adults, alongside a smaller but growing body of literature supporting Tai Chi as a non-pharmacologic intervention for both pain and social well-being. However, only a limited number of studies (n = 2–3) simultaneously evaluated both chronic pain and loneliness or social isolation outcomes within Tai Chi interventions, highlighting an important gap in the literature.

### 4.1. Which Form of Tai Chi Is Best, and for Whom?

An open area of research would be to compare the efficacy of different Tai Chi forms for managing specific chronic pain conditions. In our research, we found that both the Yang and Sun forms were popular among individuals with chronic pain, but no studies compared outcomes among groups practicing different forms of Tai Chi. For example, specific recommendations for individuals living with fibromyalgia, complex regional pain syndrome, osteoarthritis or rheumatoid arthritis, myasthenia gravis, and multiple sclerosis, considering physical limitations along with times of day when pain flares are more common to be worse (morning for fibromyalgia versus evening for osteoarthritis, myasthenia gravis, and multiple sclerosis) would be helpful.

Socialization is particularly important for quality of life among these cohorts. Research indicates that loneliness among people living with fibromyalgia is tied to higher rates of anxiety and depression [38,39]. High prevalence of loneliness has also been found among populations affected by complex regional pain syndrome [40,41], various types of arthritis [42], and multiple sclerosis [43]. Yet classes geared towards general audiences might not be appropriate for these individuals.

### 4.2. Addressing Co-Morbid Conditions for Persons Living with Chronic Pain

The feasibility of utilizing Tai Chi to address comorbid conditions is significant, since a large percentage of individuals diagnosed with non-specific chronic pain live with other chronic conditions. A retrospective review of medical records for 2431 older adults (aged 65+) found that, among the 493 with chronic pain, most had comorbid disorders (mean: 2 comorbidities per chronic pain patient), with the most frequently reported conditions including osteoarthritis, obstructive sleep apnea, anxiety, and depression [43]. Comorbidities add both complexity and expense to pharmacotherapeutic strategies, increasing the desirability of lifestyle interventions.

Tai Chi’s focus on deep diaphragmatic breathing may help with self-management for persons with chronic asthma and COPD [15]. In a small randomized controlled trial, researchers compared 12 weeks of Tai Chi instruction to usual care for patients diagnosed with COPD. Patients in the intervention group had improved self-rated quality of life and non-significant improvements in the 6 min walk test compared to the control group [44], with generally good adherence to the exercise protocol. A more recent two-arm randomized clinical trial on patients with moderate-to-severe COPD found improvements in depression and anxiety scores among members of the Tai Chi group, as well as reductions in symptom exacerbation rates [45]. 

Finally, Tai Chi can reduce stress and improve sleep quality among older adults. A quasi-experimental study among 60 older adults aged 60–80 found significant stress reduction in the experimental group following a 14-day Tai Chi intervention [46]. A randomized controlled trial at two community sites comparing a 25-week Tai Chi intervention with health education found significant improvements in sleep quality, duration, efficiency, and sleep disturbance in the experimental group versus the control group [47]. A systematic review and meta-analysis on the use of Tai Chi for improving sleep quality, anxiety and depression found statistically significant improvements in all three measures [48].

### 4.3. Controlling the Cost of Chronic Pain

Chronic pain represents a substantial and growing global burden, affecting approximately 20% of the population and contributing to significant healthcare and societal costs [49]. In the United States alone, annual costs have been estimated at over $700 billion, driven by both medical expenditures and lost productivity [50]. Although evidence-based medical and behavioral interventions for chronic pain exist, many require ongoing clinical services, specialized training, or repeated patient–provider interactions, which may limit long-term accessibility and sustainability, particularly for older adults and individuals living in low- and middle-income settings.

Findings from our review suggest that community-based, non-pharmacologic interventions such as Tai Chi may offer a promising complementary approach to addressing this burden. Several included studies reported improvements in pain outcomes, social connectedness, or quality of life following Tai Chi participation. In contrast to many clinic-based interventions, Tai Chi can be delivered in community settings at relatively low cost and may support both physical activity and social engagement simultaneously. Additional studies outside the present review have also reported associations between community Tai Chi participation and lower healthcare utilization [51], potentially reflecting broader improvements in physical and psychological well-being [52]. As such, scalable interventions that address both the physical and social dimensions of chronic pain may be particularly valuable for aging populations and resource-limited healthcare systems.

## 5. Limitations

Given the complexity of primary chronic pain and the prevalence of comorbidities, particularly among older adults, there is no optimal therapeutic strategy. This narrative review proposes a single pathway that is relatively low cost and widely available in community settings. The first author performed the initial literature search under the guidance of a university science librarian. To reduce the risk of bias, the second author reviewed the initial search for relevance and quality of extracted studies and made revisions as necessary. Finally, this narrative review is not a complete mapping of the evidence, but rather an attempt to utilize recent, high-quality research studies to support the utilization of Tai Chi as a mind–body intervention for ameliorating subjectively rated loneliness, social isolation, and primary chronic pain among older adults.

## 6. Conclusions

Both non-specific chronic pain and subjectively rated loneliness, with or without social isolation, are common co-occurrences among older adults, the population bearing the greatest burden of chronic disease globally. The rising prevalence and cost of treating primary chronic pain require urgent attention because of their effects on quality of life and the growing economic strain placed on healthcare systems worldwide. The global population of adults aged 60 years and older is projected to reach 2.1 billion by 2050, double the number reported in 2015 [53], with approximately 80% living in low- and middle-income countries. These demographic trends highlight the need for scalable, accessible, and sustainable approaches to chronic pain management that can be implemented beyond traditional clinical settings.

Low-cost, community-based approaches to chronic pain management will likely become increasingly important as healthcare systems respond to the growing burden of aging-related chronic disease. Tai Chi instruction in community settings provides opportunities for older adults to engage in physical activity while strengthening social connectedness and reducing subjectively rated loneliness. In addition, evidence supports the potential benefits of Tai Chi for improving self-reported chronic pain symptoms and related aspects of psychological well-being. Because Tai Chi can be delivered in group settings with relatively limited infrastructure and low ongoing cost, it may represent a particularly valuable intervention for resource-constrained healthcare systems and underserved populations. Given the high prevalence of comorbidities among older adults with chronic pain, Tai Chi’s reported benefits for stress, anxiety, depression, obstructive sleep apnea, arthritis, and chronic respiratory disease further support its potential role as a broadly applicable population health intervention.

## Figures and Tables

**Table 1:** Study Characteristics: Location, Design, and Participant Demographics.

Author (Year)	Country	Study Design	Sample Size (N)	Age (Mean/Range)	% Female	Population Notes
Bloomberg et al. (2025) [10]	United Kingdom	Longitudinal	7336	≥50	54.60%	Predominantly White
Brown et al. (2026) [20]	United States	Cross-sectional	82	Mean 50.5	68.30%	Native American sample
Camacho et al. (2024) [9]	United States	Cross-sectional	2706	≥50	NR	Racially diverse sample
Chan et al. (2017) [16]	China	Pilot study	48	≥60	NR	Community-based
Chen et al. (2025) [21]	Japan	Non-randomized controlled trial	84	≥60	59.50%	Exercise intervention
Dagnino & Campos (2022) [22]	Not specified	Narrative review	N/A	Older adults	N/A	Pain management review
Gillsjö et al. (2021) [23]	Sweden	Qualitative	20	72–79	70.00%	Interviews
Han et al. (2025) [8]	China	Cohort (CHARLS)	3109	≥60	NR	National longitudinal dataset
Hong et al. (2024) [24]	United States	Prospective longitudinal	13,365	Mean 69	NR	Large aging cohort
Hosseini et al. (2025) [25]	Iran	Cross-sectional	1675	Mean 69.7	45.40%	Community-dwelling
Koren et al. (2021) [26]	Not specified	Systematic review	10 studies	≥60	N/A	Mixed methods
Kotwal et al. (2021) [1]	United States	Cross-sectional	5976	≥50	50.40%	End-of-life population
Lee et al. (2020) [27]	United States	Qualitative	18	≥65	61.10%	Tai Chi intervention
Lim et al. (2024) [28]	Not specified	Systematic review and meta-analysis	22 RCTs	Pre-frail older adults	N/A	Exercise interventions
Maité et al. (2025) [29]	Belgium	Prospective (2-wave)	332	≥50	81.60%	Chronic pain population
Matsuda et al. (2025) [30]	Japan	Prospective cohort	4709	Mean 73.8	56.40%	Community-dwelling
Noguchi et al. (2023) [11]	Japan	Cross-sectional	21,463	≥65	51.60%	Large population study
Straus et al. (2022) [31]	United States	Cross-sectional	4069	Mean 62	9.80%	U.S. veterans
Tang et al. (2019) [32]	Not specified	Systematic review	10 studies	≥65	N/A	Non-pharmacologic interventions
Weber et al. (2020) [33]	Not specified	Systematic review and meta-analysis	37 RCTs (3224 participants)	≥60	N/A	Tai Chi/Qigong
Wong et al. (2024) [34]	China	Mixed methods	4153	Mean 75.3	82.70%	Community-based
Yang et al. (2024) [35]	China	Cross-sectional	1087	≥60	49.60%	Mind–body exercise
You et al. (2023) [17]	United States	Narrative review	N/A	Older adults	N/A	Tai Chi and pain
You et al. (2020) [36]	United States	Exploratory	40	Mean 74	42.50%	Biomarker study
Zheng et al. (2017) [37]	China	Qualitative	20	Mean 62	70.00%	High-risk stroke population

NR = Not Reported; N/A = Not Applicable (e.g., reviews).

**Table 2:** Study Focus, Key Variables, and Main Findings.

Author (Year)	Study Focus	Key Variables	Main Findings
Bloomberg et al. (2025) [10]	Pain and loneliness trajectory	Loneliness, depression, chronic pain	Loneliness increased before and after pain onset; depression rose sharply at onset and then stabilized
Brown et al. (2026) [20]	Social support and pain	Emotional support, instrumental support, pain intensity/interference	Social isolation associated with higher pain interference; instrumental support buffered pain effects
Camacho et al. (2024) [9]	Loneliness and pain	Loneliness, pain presence	Greater loneliness associated with higher likelihood of reporting pain
Chan et al. (2017) [16]	Tai Chi and social outcomes	Tai Chi/Qigong, loneliness, social support	Tai Chi participants reported reduced loneliness and improved social support
Chen et al. (2025) [21]	Tai Chi intervention	Tai Chi vs. resistance exercise, pain	Tai Chi significantly improved subjective pain following 12-week intervention
Dagnino & Campos (2022) [22]	Pain management strategies	Chronic pain, non-pharmacologic therapies	Tai Chi identified as effective non-pharmacologic modality for pain management
Gillsjö et al. (2021) [23]	Pain and social experience	Chronic pain, loneliness, social isolation	Pain experiences reinforced by loneliness, stigma, and lack of validation
Han et al. (2025) [8]	Pain and social factors	Chronic pain, loneliness, social isolation	Loneliness and isolation associated with increased chronic pain over time
Hong et al. (2024) [24]	Health and loneliness	Self-rated health, anxiety, purpose, pain	Poor physical and psychological health predicted greater loneliness
Hosseini et al. (2025) [25]	Pain and comorbidities	Chronic pain, sleep, depression, cognition, isolation	Chronic pain associated with poor sleep, depression, cognitive impairment, and living alone
Koren et al. (2021) [26]	Tai Chi and social support	Tai Chi, social isolation, social support	Majority of Tai Chi interventions improved perceived social support
Kotwal et al. (2021) [1]	Epidemiology of isolation	Social isolation, loneliness	High prevalence of isolation and loneliness; associated with socioeconomic and functional factors
Lee et al. (2020) [27]	Tai Chi and pain (qualitative)	Tai Chi, chronic low back pain	Mixed pain outcomes; improved motivation, relaxation, and social support
Lim et al. (2024) [28]	Exercise and frailty	Multimodal exercise, Tai Chi, frailty	Exercise improved frailty status; frequency predicted functional gains
Maité et al. (2025) [29]	Pain and stigma	Chronic pain, stigma, social isolation	Pain associated with stigma, mental distress, and increased isolation
Matsuda et al. (2025) [30]	Pain, isolation and disability	Chronic pain, social participation	Pain and isolation jointly increased risk of disability
Noguchi et al. (2023) [11]	Loneliness and back pain	Loneliness, social isolation, low back pain	Loneliness associated with back pain; combined isolation and loneliness had synergistic effect
Straus et al. (2022) [31]	Loneliness and comorbidity	Loneliness, chronic pain, mental health	Loneliness strongly associated with chronic pain and psychiatric comorbidities
Tang et al. (2019) [32]	Non-pharmacologic pain care	Tai Chi, Qigong, acupuncture, guided imagery	Mind–body interventions effective in reducing pain in older adults
Weber et al. (2020) [33]	Tai Chi and QoL	Tai Chi/Qigong, QoL, depression, sleep	Tai Chi improved QoL, reduced depression, improved sleep and social engagement
Wong et al. (2024) [34]	Mental health and treatment response	Anxiety, loneliness, cognition, pain	Loneliness and psychological factors predicted poorer treatment response
Yang et al. (2024) [35]	Mind–body exercise and QoL	Tai Chi, social support, resilience	Social support and resilience mediated relationship between exercise and QoL
You et al. (2023) [17]	Tai Chi and pain	Tai Chi, multisite pain	Tai Chi reduced pain and improved physical and cognitive function
You et al. (2020) [36]	Mechanisms of Tai Chi	Tai Chi, beta-endorphins, inflammation	Tai Chi reduced beta-endorphins, suggesting enhanced endogenous pain modulation
Zheng et al. (2017) [37]	Tai Chi (qualitative outcomes)	Tai Chi, pain, sleep, relationships	Participants reported improved pain, sleep, emotional well-being, and social relationships

## Data Availability

No new data were created or analyzed in this study. Data sharing is not applicable to this article.
